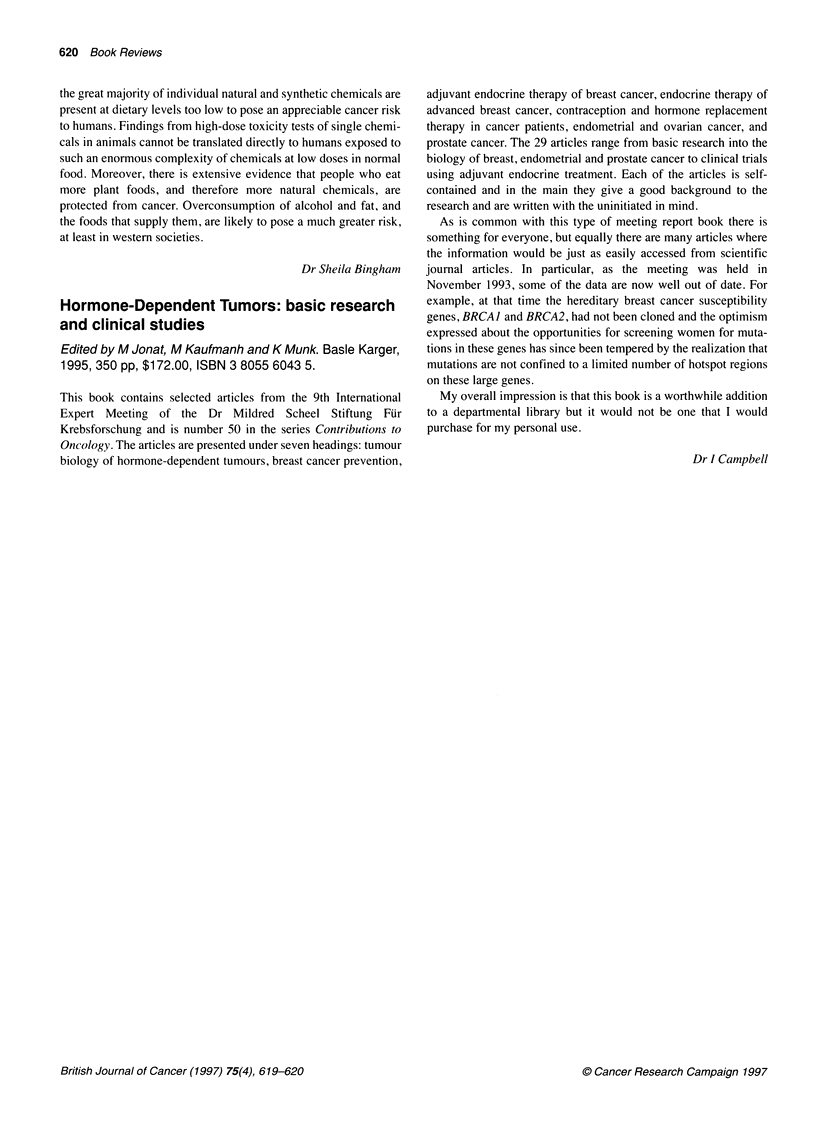# Hormone-Dependent Tumors: basic research and clinical studies

**Published:** 1997

**Authors:** I Campbell


### Hormone-Dependent Tumors: basic research and clinical studies

*Edited by M Jonat, M Kaufmanh and K Munk.* Basle Karger, 1995, 350 pp, $172.00, ISBN 3 8055 6043 5.

This book contains selected articles from the 9th International Expert Meeting of the Dr Mildred Scheel Stiftung Für Krebsforschung and is number 50 in the series *Contributions to Oncology*. The articles are presented under seven headings: tumour biology of hormone-dependent tumours, breast cancer prevention, adjuvant endocrine therapy of breast cancer, endocrine therapy of advanced breast cancer, contraception and hormone replacement therapy in cancer patients, endometrial and ovarian cancer, and prostate cancer. The 29 articles range from basic research into the biology of breast, endometrial and prostate cancer to clinical trials using adjuvant endocrine treatment. Each of the articles is self-contained and in the main they give a good background to the research and are written with the uninitiated in mind.

As is common with this type of meeting report book there is something for everyone, but equally there are many articles where the information would be just as easily accessed from scientific journal articles. In particular, as the meeting was held in November 1993, some of the data are now well out of date. For example, at that time the hereditary breast cancer susceptibility genes, *BRCA1* and *BRCA2*, had not been cloned and the optimism expressed about the opportunities for screening women for mutations in these genes has since been tempered by the realization that mutations are not confined to a limited number of hotspot regions on these large genes.

My overall impression is that this book is a worthwhile addition to a departmental library but it would not be one that I would purchase for my personal use.

*Dr I Campbell*

*© Cancer Research Campaign 1997*